# Atomistic aspects of ductile responses of cubic silicon carbide during nanometric cutting

**DOI:** 10.1186/1556-276X-6-589

**Published:** 2011-11-11

**Authors:** Saurav Goel, Xichun Luo, Robert L Reuben, Waleed Bin Rashid

**Affiliations:** 1 School of Engineering and Physical Sciences, Heriot Watt University, Edinburgh, EH144AS, Scotland, UK

**Keywords:** ductile regime nanometric cutting, silicon carbide, diamond tool, tool wear

## Abstract

Cubic silicon carbide (SiC) is an extremely hard and brittle material having unique blend of material properties which makes it suitable candidate for microelectromechanical systems and nanoelectromechanical systems applications. Although, SiC can be machined in ductile regime at nanoscale through single-point diamond turning process, the root cause of the ductile response of SiC has not been understood yet which impedes significant exploitation of this ceramic material. In this paper, molecular dynamics simulation has been carried out to investigate the atomistic aspects of ductile response of SiC during nanometric cutting process. Simulation results show that cubic SiC undergoes *sp^3^-sp^2 ^*order-disorder transition resulting in the formation of SiC-graphene-like substance with a growth rate dependent on the cutting conditions. The disorder transition of SiC causes the ductile response during its nanometric cutting operations. It was further found out that the continuous abrasive action between the diamond tool and SiC causes simultaneous *sp^3^-sp^2 ^*order-disorder transition of diamond tool which results in graphitization of diamond and consequent tool wear.

## Introduction

Silicon carbide (SiC) is a promising ceramic material suited for advanced neural interfaces, packaging for long-term implantation, microfabricated neural probe as well as for semiconductor devices used in severe environments, such as in military aircraft, combat vehicles, power generation, and petrochemical industries [1]. SiC is a very hard substance (9 to 9.5 on Mohs scale) having comparable hardness to the hardest material known as diamond (10 on Mohs scale). The unique blend of properties possessed by SiC which makes it suitable for various MEMS, bio-medical, and other applications can be summarised in the form of Table 1[2].

**Table 1:** Commercial applications of SiC [2]

Serial number	Properties of SiC	Applications	Realisation
1	High sublimation temperature	High temperature transducer elements	High temperature sensor diaphragms and resonators
2	Large band gap	High temperature electronics	Sensors for smart engines
			On chip signal conditioning
3	Low wear and high hardness	Enhanced durability/operation	Coated mechanical contacts
			Microfabricated bearings
4	Chemically inert	Stable in harsh environments	Valve/pumps for corrosives
			Flow sensors for acids

Moreover, SiC is also capable of meeting the requirements of operation in hostile environments (up to 873 K) where conventional silicon-based electronics (limited to 623 K) cannot function. The National Aeronautics and Space Administration agency, NASA, has recently been making efforts to develop SiC as future material for advanced semiconductor electronic device applications [3].

Single-point diamond turning (SPDT) is now an established ultra-precision machining process to manufacture free-form shapes and mirror-finished machined surfaces [4,5]. SPDT was established by exploiting a so called "brittle-ductile transition" phenomenon which has made various brittle materials, amenable to ultra-precision machining using a diamond cutting tool [6,7]. Investigations on exploring silicon carbide as a diamond-turnable material are thus of scientific and technological interest.

Experimental investigation on the feasibility of ductile regime machining of SiC through SPDT was first reported in 2005 [8]. Common believe on machining mechanism of SiC has been that a nanoscale undeformed chip thickness compounded with slow feed rate helps to achieve high-pressure phase transformations (HPPT) which causes ductile responses from this brittle material [9,10]. However, no such evidence of HPPT during nanometric cutting of SiC has been reported yet and the root cause of ductile response of SiC is still unknown. Molecular dynamics (MD) simulation results have been successful in the past to address number of problems concerning the nanometric cutting processes of brittle materials like silicon [11-14].

In this paper, Tersoff potential energy function [15] was used in the MD simulation to elucidate the atomistic mechanism underlying the ductile responses from the cubic SiC during nanometric cutting. Resorting to the simulation results, a theory has been presented and discussed.

## MD simulation

### MD simulation model

**Figure 1:**
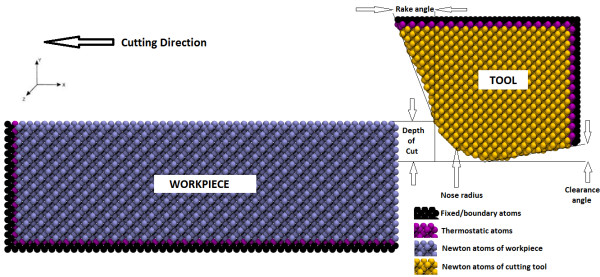
**Schematic of MD simulation model**.

A schematic diagram of the nanometric cutting simulation model is shown in Figure 1. In the simulation model, the single-crystal diamond cutting tool has been modelled as a deformable body. Accordingly, both the work piece and the cutting tool are divided into three different zones: Newton atoms, thermostatic atoms, and boundary atoms. The boundary atoms are unaffected during the simulation and remain fixed in their initial lattice positions, serving to reduce the boundary effects and maintain the symmetry of the lattice.

The motions of the atoms in the Newton and thermostatic zones are assumed to follow the classical Newton's second law of motion which can be computed from the interatomic forces produced by the interaction as follows:

(1) $$a_{ix}=\frac{F_{ix}}{m_{i}}=\frac{d^{2}x_{i}}{dt^{2}}\;F_{ix}=-\frac{dV}{dx_{i}}$$

where *a_ix _*represents the *i*th atom's acceleration in the *x *direction, *m_i _*is the mass of the *i*th atom, *F_ix _*is the interaction force acting on the *i*th atom by the *j*th atom in the *x *direction, *x*_i _indicates the *i*th atom's *x*-coordinate and *V *is the potential energy.

During realistic machining operation, the energy from plastic deformation in the primary shear zone and friction at the tool-chip interface transforms to cutting heat, which is carried away by chips and lubricants. The motion of the thermostatic atoms is therefore scaled using the velocity scaling factor (shown in equation 2) to incorporate the dissipation of heat in the simulation.

(2) $$\mathrm{Velocity}\;\mathrm{scaling}\;\mathrm{factor}=\sqrt{\frac{\text{3}Nk_{\text{b}}T}{\underset{i}{\sum }m_{i}v_{i^{\text{2}}}}}$$

The temperature of atoms during the machining simulation can be calculated using the conversion between the kinetic energy (K.E.) and temperature as shown in Eq. 3.

(3) $$\frac{1}{2}\underset{i}{\sum }m_{i}{v_{i}}^{2}=\frac{3}{2}Nk_{\text{b}}T$$

where *N *is the number of atoms, *v_i _*represents the velocity of *i*th atom, *k*_b _is the Boltzmann constant which is equal to 1.3806503 × 10^−23 ^J/K and *T *represents the temperature on atoms. However, the instantaneous fluctuations in K.E. of atoms could be very high so K.E. should be averaged (time and/or spatial) over few timesteps to be converted into equivalent temperature. It shall be noted here that the movement of the tool will also be a contributor to the kinetic energy so the tool displacement was accordingly subtracted before equivalent temperature conversion.

### Selection of potential energy function

The interaction potential function governs the accuracy of a molecular dynamic simulation which in turn defines the reliability of simulation results. In this paper, Tersoff potential energy function [15] was used in the simulation for accurate description of the SiC mixture. Being a three-body potential function, Tersoff function is suitable to describe covalent interactions of Si and C atoms. Therefore, it was used to describe Si-Si, C-C, and Si-C interactions for interactions in and among the tool and workpiece as follows:

(4) $$E=\underset{i}{\sum }Ei=\frac{1}{2}\underset{i\neq j}{\sum }V_{ij},V_{ij}=fc\left(r_{ij}\right)\left[f_{R}\left(r_{ij}\right)+b_{ij}f_{A}\left(r_{ij}\right)\right]$$

(5) fR(rij)=Aij exp(-λijrij),fA(rij)=-Bij exp(-μijrij)

(6) $$fc\left(r_{ij}\right)=\left\{\begin{array}{c} 1\\ \frac{1}{2}+\frac{1}{2}\cos\left[\pi \frac{r_{ij}-R_{ij}}{S_{ij}-R_{ij}}\right]\\ 0 \end{array}\;\begin{array}{c} r_{ij}<R_{ij}\\ S_{ij}>r_{ij}>R_{ij}\\ r_{ij}>S_{ij} \end{array}\right)$$

(7) $$b_{ij}=\chi_{ij}{\left(1+{\beta_{i}}^{ni}{\zeta_{ij}}^{ni}\right)}^{-1∕2{n_{i}}_{}},\zeta_{ij}=\underset{k\neq i,j}{\sum }fc\left(r_{ik}\right)\omega_{ik}g\left(\theta_{ijk}\right)$$

(8) $$g\left(\theta_{ijk}\right)=1+\frac{{c_{i}}^{2}}{{d_{i}}^{2}}-\frac{{c_{i}}^{2}}{\left[{d_{i}}^{2}+h_{i}-\cos\theta_{ijk}\right]}$$

where *E_i _*is the site energy-the sub-function, *V_ij _*describes the energy between two atoms (*i *and *j*), (*i*, *j*, and *k) *label the atoms of the system, *f*_R _represents a repulsive pair potential, *f*_A _represents an attractive pair potential, *f*_C _represents a smooth cut-off function to limit the range of the potential, *r*_*ij *_is the length of the *i-j *bond, *b_ij _*is the bond order term, *ζ_ij _*counts the number of other bonds to atom *i *besides the *i-j *bond and *θ_ijk _*is the bond angle between the bonds *i-j *and *i-k*. Here the indices *ij *represents the atom species. The mixing parameters between the two atomic species can be obtained from the following mixing rules:

(9) $$\lambda_{ij}=\frac{\lambda_{i}+\lambda_{j}}{2}$$

(10) $$\mu_{ij}=\frac{\mu_{i}+\mu_{j}}{2}$$

(11) $$A_{ij}=\sqrt{A_{i}A_{j}}$$

(12) $$B_{ij}=\sqrt{B_{i}B_{j}}$$

(13) $$R_{ij}=\sqrt{R_{i}R_{j}}$$

(14) $$S_{ij}=\sqrt{S_{i}S_{j}}$$

Parameter *χ*_*ij *_determines the attractive interactions between two atoms. The potential function parameters used in the study have been listed in Table 2.

**Table 2:** Tersoff potential parameters [15]

	Si-Si	C-C	Si-C
*A *(eV)	1,830.8	1,544.8	1,681.731
*B *(eV)	471.18	389.63	432.154
*λ *(Å^−1^)	2.4799	3.4653	2.9726
*μ *(Å^−1^)	1.7322	2.3064	2.0193
* β*	1.1 × 10^−6^	4.1612 × 10^−6^	-
* n*	0.78734	0.99054	-
* c*	1.0039 × 10^5^	19981	-
* d*	16.217	7.034	-
* h*	−0.59825	−0.33953	-
*R *(Å)	2.7	1.8	2.20454
*S *(Å)	3	2.1	2.50998
*χ*_Si-C_	1.0086		

Large-scale atomic/molecular massively parallel simulator software [16] was used to perform the simulation.

### Calculation of equilibrium lattice parameter

Using inappropriate lattice parameter will affect the total energy content of the system which may lead to lots of thermal vibrations during equilibration process. Thus, the resulting fluctuations will alter the machining parameters like undeformed chip thickness, nose radius, etc. to a large extent during energy minimization which will produce erroneous simulation results. Goel et al. [17,18] have recently suggested to use the equilibrium lattice parameters to represent realistic MD simulation.

Accordingly, in the current work, equilibrium lattice parameters as shown in Table 3 were used as an input to the MD simulation in order to obtain meaningful simulation results [19,20].

**Table 3:** Comparison of lattice parameters obtained through experiment and simulation

Material	Experimental known lattice parameter at 300 K(Å)	Calculated equilibrium lattice parameter at 300 K (Å)
β-SiC (cubic)	4.3596 [19]	4.28
Diamond	3.56683 [20]	3.56

### MD simulation setup

MD simulation model of single-crystal SiC and diamond was built using the periodic boundary conditions along the *z *direction. This was followed by an energy minimization to avoid overlaps in the positions of the atoms. The simulation model was equilibrated to 300 K under the microcanonical (NVE) ensemble and the initial velocities of the atoms were assigned in accordance with a Maxwell-Boltzmann distribution. During the equilibration process, the trajectories must not be used to compute any properties as the potential energy continues to convert to kinetic energy or vice-versa. This causes the temperature to fluctuate until it becomes stationary. Once sufficient time has been given for equilibration, velocity scaling is removed and the system then follows NVE dynamics. Different variables which were used in the current simulation model have been listed in Table 4.

**Table 4:** Variables used in the MD simulation model

Dimensions of SiC workpiece	14.2624 × 4.6353 × 5.4845 nm
Numbers of β-SiC atoms in the workpiece	38,324
Numbers of carbon atoms in the cutting tool	27,373
Tool nose radius	2.2974 nm
Undeformed chip thickness	1.3128 nm
Tool rake and clearance angle	-25° and 10°
Workpiece machining surface	(010)
Tool orientation and cutting direction	Cubic and <100>
Equilibration temperature	300 K
Cutting velocity	100 m/s
Timestep	0.5 fs

**Figure 2:**
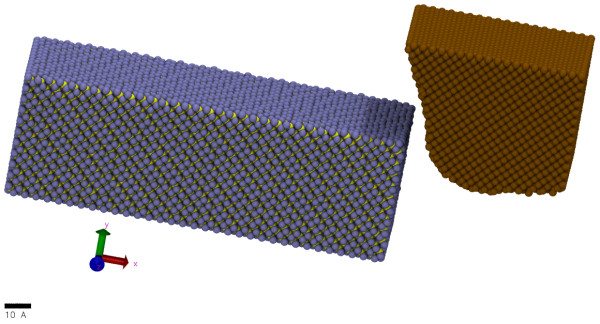
**Orthographic view of model after relaxation**.

A model output from the MD simulation after relaxation has been shown in Figure 2 where, ice blue colour^a ^and yellow colour correspond to silicon atoms and carbon atoms in the cubic SiC, respectively. The ochre colour represents carbon atoms in the diamond tool. Visual Molecular Dynamics (VMD) [21]^b ^along with Ovito [22]^c ^were used for the enhanced visualisation of the atomistic data.

**Figure 3:**
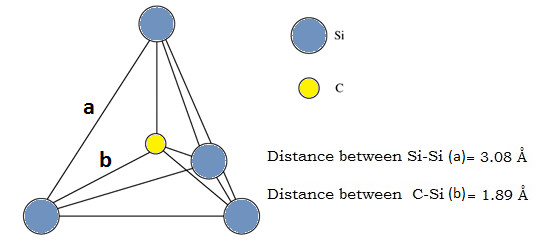
**Lattice structure of cubic SiC**.

As shown in Figure 3, SiC has a tetrahedral molecular geometry which has a central carbon atom surrounded at equidistance by four silicon(Si) atoms or vice-versa and thus bond angles are cos^−1^(−1/3) ≈ 109.5° between them. A tetrahedral geometry can however, be distorted by increasing the bond angles. This distortion of lattice structure and consequent increase in the bond angle is accompanied by the changes in the bond length. This phenomenon is called planarization which causes flattening of tetrahedron structure due to increment in bond angle. During MD simulation, a similar phenomenon was observed which has been discussed further in the subsequent sections.

## Results and discussions

### Observations from MD simulation results

**Figure 4:**
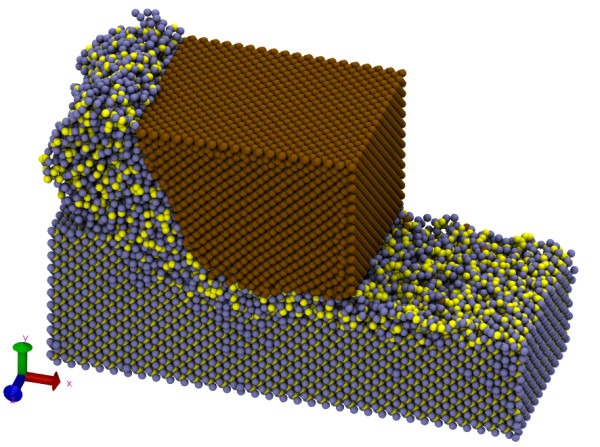
**Chip morphology of β-SiC (cubic) during the nanometric cutting process**.

Figure 4 shows the snapshot from the MD simulation after 10 nm of tool advance. The work piece shown in Figure 4 has Si-terminated surfaces having silicon atoms at the edge of the workpiece. The nature of SiC is extremely brittle and hence surface roughness on the machined surface of SiC in Figure 4 appears to be relatively higher as has been observed during the experimental work [23].

Figure 4 also represents the chip morphology of β-silicon carbide (cubic) during the nanometric cutting process against a deformable diamond tool. It can be seen from Figure 4 that the cutting chips are curly shaped, which suggests that material removal is occurring in ductile regime by deformation rather than fracture. During the machining process, a few carbon atoms from the diamond tool deformed and are separated from the tool which can be seen on the machined surface of SiC.

### Temperature during the machining process

**Figure 5:**
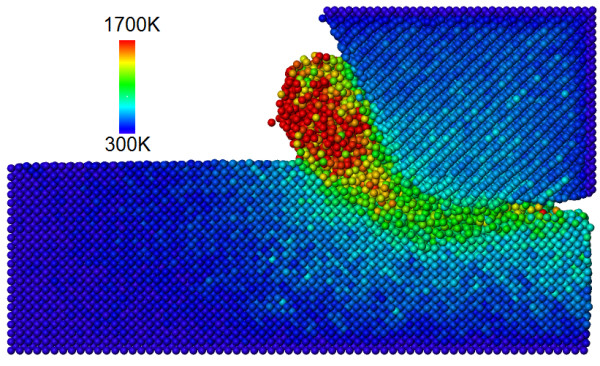
**Temperature distribution on the workpiece and the cutting tool after 4 nm of cut**.

Figure 5 represents the temperature distribution on the workpiece and the cutting tool during nanometric cutting after the cutting tool has advanced by 4 nm. It can be seen that the maximum temperature in the primary shear zone of the workpiece approaches to a value of 1,700 K. The high temperature in the primary shear zone on SiC during its nanometric cutting is closer in magnitude to the enabling temperature(1,400°C) for the formation of SiC-graphene on either Si- or C-terminated SiC surfaces as reported [24]. Accordingly, it is plausible to state that high temperature ignites the formation of SiC-graphene-like substance during the nanometric cutting operation of SiC. SiC-graphene being much weaker and slippery compared to cubic SiC causes ductile responses in the cutting zone. The formation of SiC graphene from cubic SiC is essentially an outcome of *sp^3^-sp^2 ^*order disorder transition. In order to ensure the presence of a substance like SiC-graphene during cutting, angular distribution functions, and radial distribution functions of SiC before and during the cutting were plotted which have been elaborated in subsequent sections.

### Angular distribution function of SiC during cutting

**Figure 6:**
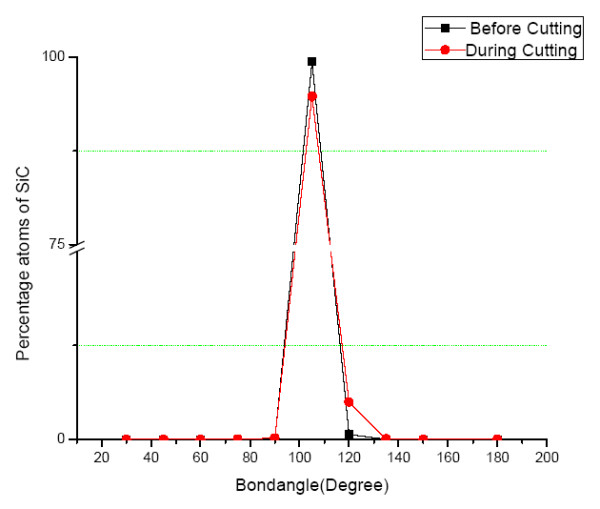
**Angular distribution function of SiC before and during cutting**.

**Figure 7:**
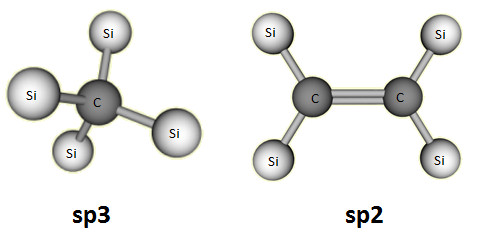
***sp^3^-sp^2 ^*hybridisation**.

Figure 6 represents angular distribution function of SiC workpiece obtained from the simulation before and during the cutting. It is evident from Figure 6 that before cutting, the peak of angular distribution function of SiC is visible at a bond angle of 109.5° which confirm the perfect *sp^3 ^*tetrahedral bonding structure of SiC. During cutting, the bond angle of around 5% of atoms reduces at the bond angle of 109.5° with a corresponding increase and a small peak at a value of 120°. A change in value of bond angle from 109.5° to 120° is a strong indication of *sp^3^-sp^2 ^*hybridisation in the manner shown in Figure 7.

As shown in Figure 7, the *sp^3 ^*hybridisation has a tetrahedral geometry with a bond angle of 109.5°. However, when this tetrahedral geometry is distorted the bond angle changes to 120° and results in planarization and consequent *sp^2 ^*hybridisation. This *sp^3^-sp^2 ^*transition occurs due to the abrasive action between diamond cutting tool and SiC workpiece. Thus, a change in bond angle from 109.5° to 120° obtained through angular distribution function is an indication of *sp^3^-sp^2 ^*order-disorder transition and transformation of cubic silicon carbide to SiC-graphene-like substance.

### Radial distribution function and relative tool wear

The radial distribution functions (RDF), *g*(*r*), also called pair distribution functions or pair correlation functions, are the primary linkage between macroscopic thermodynamic properties and intermolecular interactions.

**Figure 8:**
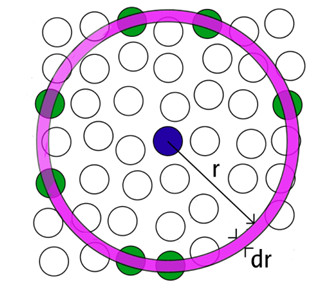
**Space discretization for the evaluation of the radial distribution function **[25] .

**Figure 9:**
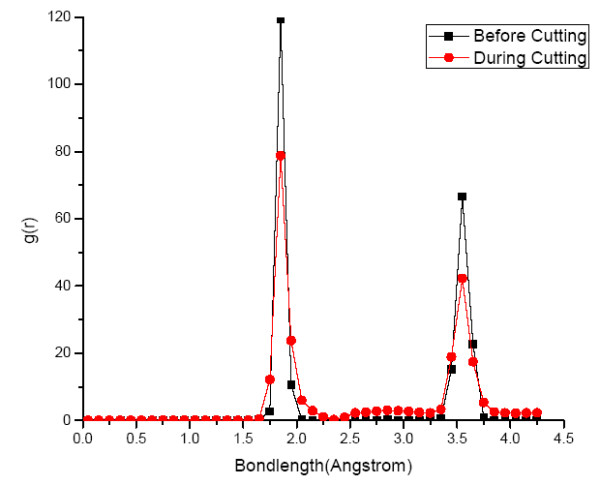
**Radial distribution function of SiC workpiece**.

As illustrated in Figure 8[25], if the atoms are distributed homogeneously in space, then the RDF, *g*(*r*), gives the probability of finding an atom in a shell dr at a distance *r *from another atom chosen as a reference point. The number of atoms dn(*r*) at a distance between *r *and *r *+ dr from a given atom is expressed as follows:

(15) $$\text{dn}\left(r\right)=\frac{N}{V}g\left(r\right)4\pi r^{2}\text{dr}$$

where *N *represents the total number of atoms, *V *is the model volume and *g*(*r*) is the radial distribution function. Thus, *g*(*r*) can be employed to observe the variation in magnitude of bond length of atoms during nanometric cutting. Accordingly, radial distribution functions of SiC workpiece and diamond tool were plotted before and during the cutting process which has been shown in Figure 9.

**Figure 10:**
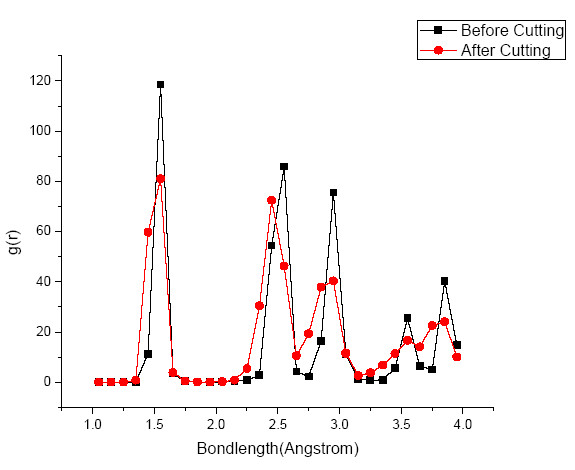
**Radial distribution function of diamond tool**.

It is evident from Figure 9 that before cutting, the first peak of RDF in SiC workpiece was visible at a bond length of 1.85 Å which is the equilibrium bond length of cubic SiC. A second small peak at a bond length of 1.9 Å was also evident which represents Si-terminated dangling bonds on the surface. During nanometric cutting, the number of atoms at a bond length of 1.85 Å decreases with corresponding major increase at a bond length of 1.9 Å while few others at a bond length of 1.75 Å. These two bond lengths of 1.9 and 1.75 Å are in excellent correlation with the reported bond length 1.87 Å [26] and 1.77 Å [27] of Si-terminated graphene and Si = C double bond length respectively. Thus, RDF reconfirms the presence of SiC-graphene-like substance during the nanometric cutting process. The high magnitude of abrasion between SiC and diamond tool also results in the *sp^3^-sp^2 ^*order-disorder transition and consequent graphitization of diamond tool but at relatively slower rate. The RDF of the diamond tool before and after the cutting is shown in Figure 10.

It is widely known that the thermal stability of diamond gets adversely effected in the environment of severe temperature [28]. This causes graphitization/*sp^3^-sp^2 ^*order-disorder transition of the diamond tool during nanometric cutting of SiC. It can be seen from Figure 10 that at timestep 0, the RDF of diamond tool shows its first peak at 1.54 Å which is the known bond length of diamond [29] while few bonds(dangling bonds) on the surface shows a small peak at 1.42 Å. During cutting, the small peak continued to grow at a bond length of 1.42 Å with corresponding decrease in the number of atoms at the bond length of 1.54 Å. The bond length of 1.42 Å is the known bond length of another stable allotrope of carbon known as graphite [29] which is much weaker than diamond due to the layered structure. Thus, *g*(*r*) confirms the graphitization of the diamond tool during SPDT operation of cubic SiC and consequent wear as earlier observed from Figure 4. The numbers of the SiC atoms in the cutting chips are more than those deformed carbon atoms from the diamond tool and this proves that the rate of *sp^3^-sp^2 ^*transition of diamond tool is relatively slower than SiC.

**Figure 11:**
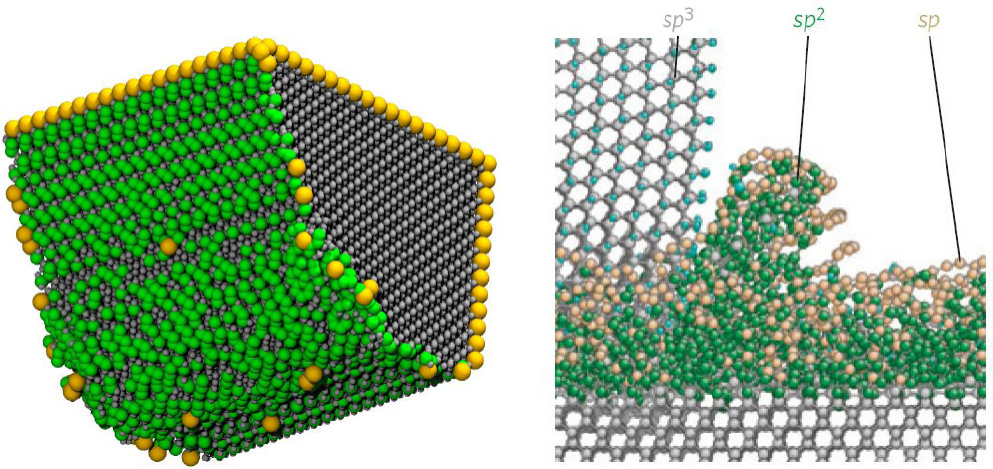
**Wear of diamond tool**. **(a) ***sp^3^*-*sp^2 ^*transition during SPDT of SiC, **(b) ***sp^3^*-*sp^2 ^*transition during polishing of diamond [30]

It thus becomes plausible to state that the abrasion between SiC and the diamond tool causes the temperature rise in the cutting zone and consequent *sp^3^-sp^2 ^*order-disorder transition of both these materials. The disorder taking place inside the diamond tool has been pictorially showed in Figure 11 where silver colour atoms represent *sp^3^*; green colour represents *sp^2 ^*while yellow colours represent *sp *arrangement. The regular arrangement of *sp^2 ^*and *sp *bonds at the tool cutting edges represents termination of bonds (dangling bonds) on the surface and sides of the cutting tool. However, the *sp^3^-sp^2^-sp *bond transition around the tool nose appears as an initiation point of graphitization of diamond tool.

The phenomenon of s*p^3^*-*sp^2 ^*order-disorder transition during SPDT of SiC also appears to be similar in nature to what occurs during polishing of diamond, which has been explained in details by Pastweka et al. [30] using MD simulation studies.

## Conclusions

The MD simulation has been used to gain extensive insights into the atomistic aspects of ductile responses of SiC during nanometric cutting operations. The following conclusions can be drawn accordingly:

During nanometric cutting, the tetrahedral bonding structure of β-SiC work material gets distorted accompanying the change of bond angle from 109.5° to 120° which represents *sp^3^-sp^2 ^*order-disorder transition of SiC.

This *sp^3^-sp^2 ^*disorder causes the formation of SiC-graphene-like substance which causes ductile response from cubic SiC.

The formation of SiC-graphene-like substance is attributed to the high temperature during the nanometric cutting which is consequent due to the abrasive action between these two ultra-hard materials.

Abrasive action also causes simultaneous *sp^3^-sp^2 ^*order-disorder transition of the diamond tool but at relatively slower rate which results in tool wear.

## Competing interests

The authors declare that they have no competing interests.

## Authors' contributions

SG did the literature review and carried out the MD simulation. WBR provided some basic inputs to the MD simulation. XL suggested the basic framework of the manuscript and also did the proof reading of the manuscript. RLR helped with the critical corrections wherever necessary. All authors read and approved the final manuscript.

## Endnotes

^a ^Readers are requested to refer to the web-based version of this article for correct interpretation of the colour legends.

^b ^Developed at the University of Illinois, USA

^c ^Developed at LLNL, USA
